# Post-vaccination Adverse Reactions After Receiving the Pfizer-BioNTech Coronavirus Disease 2019 Vaccines Among Healthcare Workers in Sapporo, Japan

**DOI:** 10.7759/cureus.23549

**Published:** 2022-03-27

**Authors:** Yoshinosuke Shimamura, Yoshiyasu Anbo, Yasushi Furuta

**Affiliations:** 1 Office of Infection Control and Prevention, Teine Keijinkai Medical Center, Sapporo, JPN

**Keywords:** pfizer-biontech, post-vaccination reactions, japan, healthcare workers, covid-19 vaccine

## Abstract

Background/objective

Although a third dose of the coronavirus disease 2019 vaccine was initiated, the reports of the post-vaccination adverse reactions after dose three from Japan were limited. We aimed to report on post-vaccination adverse reactions to the third dose of the vaccine among healthcare workers and compare the results with those after the first two doses of vaccine at a tertiary medical center in Japan.

Materials and methods

After each vaccine (Pfizer-BioNTech) administration, healthcare workers answered a Web-based questionnaire for two consecutive days regarding local and systemic adverse reactions and anaphylaxis reactions. Information about those who took antipyretics and analgesics was also collected. Data were collected using Microsoft Forms (Microsoft, Redmond, WA, USA), a web-based questionnaire software. We compared the proportions of post-vaccination adverse reactions among the three doses of vaccine using the chi-squared test.

Results

A total of 1,990 employees received the first dose in March 2021, 1,988 employees received the second dose in April 2021, and 1,748 employees received the third dose between December 2021 and January 2022. The median age was 32 years and 21% were men. Local and systemic adverse reactions were greater after dose three than those with the primary series, except for nausea and vomiting. Injected site pain, fatigue, and headache were the three most commonly reported adverse reactions throughout the three sessions. A total of four employees developed anaphylaxis reactions. Additionally, 944 and 1,016 employees reported taking antipyretics and analgesics after doses two and three.

Conclusions

The coronavirus 2019 booster vaccine was safe and well-tolerated. Clinicians should encourage the public to receive the coronavirus 2019 vaccine series.

## Introduction

Although more than 70% of the Japanese population had received two doses of the coronavirus disease 2019 (COVID-19) vaccine by the end of December 2021 [1], an increase in the incidence of COVID-19 has been observed since January 2022 [2], mainly due to the emergence of new variants of severe acute respiratory syndrome coronavirus 2 (SARS-CoV-2) and waning vaccine-elicited immunity [3-5]. In Japan, we are encountering the sixth wave of COVID-19 and the incidence of COVID-19, including reinfection cases, exceeds 100,000 per day. To date, COVID-19 vaccines manufactured by Pfizer-BioNTech, Moderna, and AstraZeneca are approved by the Japanese Ministry of Health, Labour, and Welfare. The Japanese government determined it was important to administer a third dose of a COVID-19 vaccine in December 2021. This was initially indicated for use in healthcare workers (HCWs) who had received a second dose at least five months earlier [6]. The results of many prior studies [7-10] have reported that a third dose of the COVID-19 vaccine was effective in preventing infection and reducing disease severity. As regards safety, the post-vaccination reactions after a third dose of the vaccine have been reported to be similar to those after the first two doses [11,12]; however, a knowledge gap exists because reports of reactions after dose three from Japan outside of clinical trial settings were scarce. Therefore, we aimed to report on post-vaccination reactions to the third dose of COVID-19 vaccine among HCWs and compare the results with those after the first two doses of vaccine at a tertiary medical center in Sapporo, Japan.

## Materials and methods

Study design and participants

This is a single-center cross-sectional study. Our methods are the same as those applied in our previous study [13]. All HCWs at the Teine Keijinkai Medical Center who were scheduled to receive the Pfizer-BioNTech vaccine according to the manufacturer’s instructions were included. The first dose was administered in March 2021, the second dose was administered in April 2021, and the third dose was administered between December 2021 and January 2022. The participants in this report provided their written informed consent for data collection and publication. This study was conducted in compliance with the Declaration of Helsinki. The institutional review board of Teine Keijinkai Medical Center approved the study (approval number 2-021361-00).

Data collection and measurements

Data were collected using Microsoft Forms (Microsoft, Redmond, WA, USA) anonymously to protect HCWs' privacy. After each vaccine administration, HCWs received smartphone text messages and weblink to initiate a Web-based questionnaire for two consecutive days, and they were asked questions about local reactions (injected site pain, redness, and swelling) [14], systemic reactions (headache, fatigue, myalgia, arthralgia, nausea/vomiting, chills, and fever >38 degrees Celcius) [14], and anaphylaxis reaction [15]. Information about those who took antipyretics and analgesics, including acetaminophen and nonsteroidal anti-inflammatory drugs, was also collected. Two independent reviewers (YS and YA) performed independent data extraction using a standardized data collection form to reduce measurement bias.

Statistical analyses

We compared the proportions of local and systemic post-vaccination reactions among the three doses of COVID-19 vaccine using the chi-squared test. STATA version 15.4 (Stata Corp LLC, College Station, TX, USA) was used to perform the statistical analyses. A P-value <0.05 was considered statistically significant.

## Results

**Figure 1:**
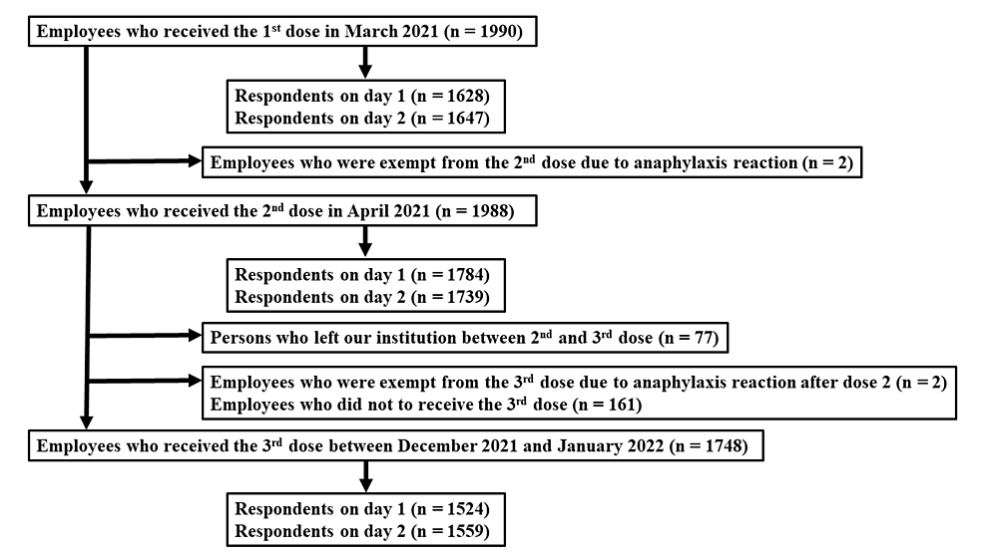
Flow chart showing the selection process of the study population.

A total of 1,990 employees received the first dose in March 2021 (the vaccination rate was 100%), 1,988 employees received the second dose in April 2022 (99%), and a total of 1748 employees received a third dose of the COVID-19 vaccine (88%) after completing the primary series between December 13, 2021, and January 14, 2022. Among those who received the third dose, a total of 1,524 (87%) individuals responded to at least one online questionnaire within two days after dose three (Figure 1).

**Figure 2:**
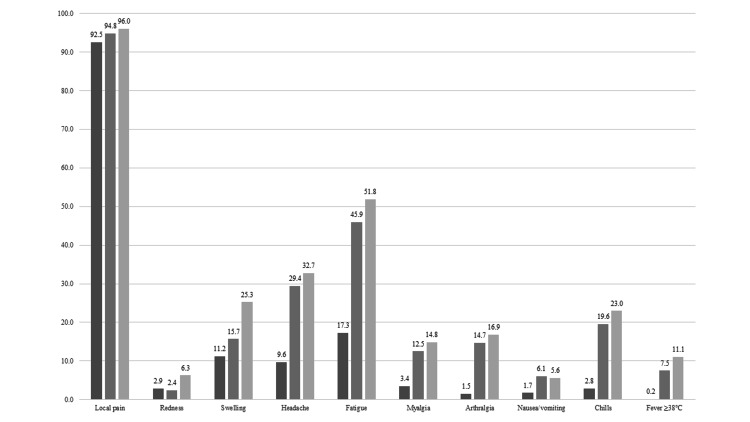
Local and systemic reactions to COVID-19 vaccine reported 1 day after vaccination. Dark gray bar, dose 1; Gray bar, dose 2, Light gray bar, dose 3.

**Figure 3:**
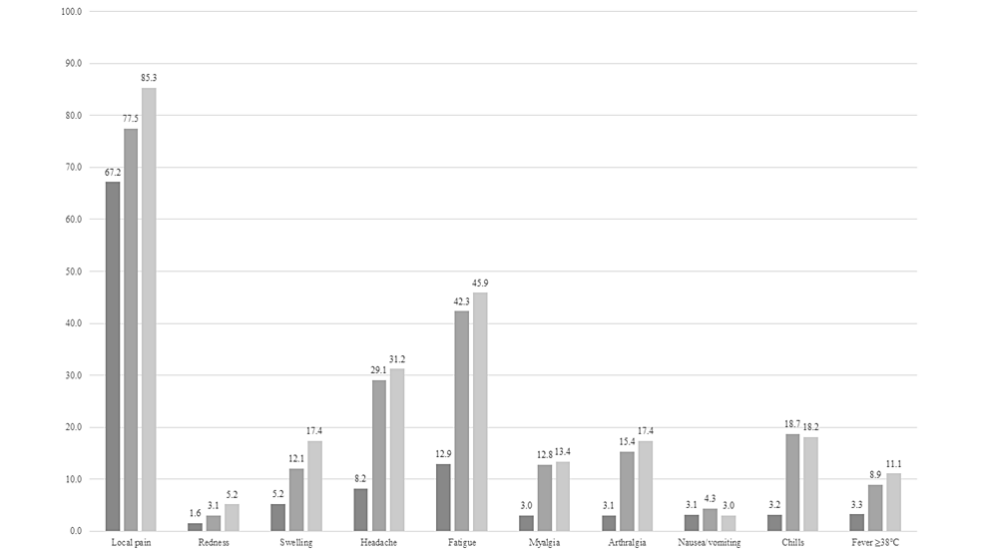
Local and systemic reactions to COVID-19 vaccine reported 2 days after vaccination. Dark gray bar, dose 1; Gray bar, dose 2, Light gray bar, dose 3.

The median age of respondents was 32 years (3.9% were 60 years old or older), and 21% were men. Figure 2 and Figure 3 show post-vaccination reactions of each dose of vaccine on day 1 and day 2. Both local and systemic reactions were greater after dose three than those with the primary series, except for nausea and vomiting. The common reported reactions at day 1 were injected site pain (92.5% after dose one, 94.8% after dose two, and 96.0% after dose three; P-value <0.05), fatigue (17.3% after dose one, 45.9% after dose two, and 51.8% after dose three; P-value <0.05) and headache (9.6% after dose one, 29.4% after dose two, and 32.7% after dose three; P-value <0.05). The proportion of post-vaccination reactions at day 2 decreased compared with that on day 1. A total of four HCWs developed anaphylaxis reactions (two persons after dose one and two persons after dose two). Additionally, 944 (48%) and 1016 HCWs (61%) reported taking antipyretics and analgesics after doses two and three, respectively. Data on the history of taking antipyretics and analgesics after dose one are missing because these were not measured.

## Discussion

Our study showed that post-vaccination reactions, both local and systemic, were reported more frequently after dose three than with the primary series, and injection site pain, fatigue, and headache were the most commonly reported reactions throughout the three sessions. Most of the reactions reached their peak one day after the vaccine administration, except for myalgia, arthralgia, and fever.

Our findings regarding the higher number of local reactions after dose three, as well as local pain, fatigue, and headache as the most common post-vaccination reactions, are in line with the results of a prior study that collected information on adverse reactions after COVID-19 vaccination using the v-safe surveillance system. The authors reported that the proportion of those experiencing local reactions, in those who received the Pfizer-BioNTech vaccine, was 71.7% after dose two; however, this increased to 74.1% after receipt of dose three [12]. However, our finding that systemic reactions were also reported more frequently after dose three, compared to that following the primary series, is in contrast with the results of the prior study [12]. One reason for this discrepancy may be related to a difference in the study population because our cohort only included immunocompetent HCWs with a smaller proportion of persons aged 60 years or older. Another reason may be related to differences in the methods of data collection. The v-safe system surveyed symptoms up to seven days after each dose of vaccine, whereas we collected information for only two consecutive days after each vaccine dose was administered due to the limited research budget. Furthermore, the discrepancy may reflect a difference in response rate; our study had ≥80% of responses after all three doses, as compared with 58.1% of the registrants in the v-safe surveillance. This might have affected the findings on post-vaccination reactions.

Our results have several implications. First, the results of this study suggest that the COVID-19 vaccine can be administered safely given that most of the post-vaccination adverse reactions were mild after all three doses of vaccine and that post-vaccination reactions are predictable and manageable. Indeed, no anaphylaxis reactions were observed after dose three. Second, our results indicated that the most of reactions are transient and resolve in a few days. These clinical implications are particularly reassuring for persons who plan to avoid the third dose due to personal safety concerns. From a research perspective, further study should be conducted to assess the association between the third dose of the Pfizer-BioNTech vaccine and the incidence of COVID-19 among HCWs in our institution, similar to a previous study conducted in Israel [16].

This study has some limitations. First, the participation in our online questionnaire was voluntary, and there might have been non-responder bias. However, the response rate in our system was much higher than that in the v-safe system. Second, the study population excluded HCWs who did not receive dose three because the decision to receive the booster was dependent on personal preference. Third, we did not assess the severity of post-vaccination reactions. It may be necessary to update our questionnaire by adding items to measure the severity of symptoms [12]. Fourth, our follow-up duration was markedly short. Longer follow-up may be required to evaluate vaccine safety more accurately. Finally, the generalizability of our findings may be limited because our study population did not include younger adolescents, children, pregnant women, elderly persons, or persons who received an additional dose from a different manufacturer.

## Conclusions

In conclusion, the results of our study showed that the COVID-19 vaccines, including the booster dose, were safe and well-tolerated. We believe this study is valuable because our findings provide information for the public, HCWs, and policymakers as we clarified the adverse reactions after the COVID-19 vaccine in the real-world setting. Based on our results, clinicians may reassure the recipients that most of these reactions are transient and resolve in several days. Finally, we, as healthcare providers, should encourage the public to receive the COVID-19 vaccines.
